# Light signaling regulates root-knot nematode infection and development via HY5-SWEET signaling

**DOI:** 10.1186/s12870-024-05356-2

**Published:** 2024-07-11

**Authors:** Bohong Wu, Xueying Jia, Wei Zhu, Yin Gao, Kefei Tan, Yuxi Duan, Lijie Chen, Haiyan Fan, Yuanyuan Wang, Xiaoyu Liu, Yuanhu Xuan, Xiaofeng Zhu

**Affiliations:** 1 Nematology Institute of Northern China, College of Plant Protection, Shenyang Agriculture University, Shenyang, China; 2 College of Biological Science and Technology, Shenyang Agriculture University, Shenyang, China; 3 College of Sciences, Shenyang Agriculture University, Shenyang, China; 4 Helongjiang Academy of Agricultural Sciences, Qiqihar, China

**Keywords:** *Arabidopsis thaliana*, Photoreceptors, *HY5*, *Meloidogyne incognita*, Sugar transporters

## Abstract

**Background:**

*Meloidogyne incognita* is one of the most important plant-parasitic nematodes and causes tremendous losses to the agricultural economy. Light is an important living factor for plants and pathogenic organisms, and sufficient light promotes root-knot nematode infection, but the underlying mechanism is still unclear.

**Results:**

Expression level and genetic analyses revealed that the photoreceptor genes *PHY, CRY*, and *PHOT* have a negative impact on nematode infection. Interestingly, ELONGATED HYPOCOTYL5 (*HY5*), a downstream gene involved in the regulation of light signaling, is associated with photoreceptor-mediated negative regulation of root-knot nematode resistance. ChIP and yeast one-hybrid assays supported that *HY5* participates in plant-to-root-knot nematode responses by directly binding to the *SWEET* negative regulatory factors involved in root-knot nematode resistance.

**Conclusions:**

This study elucidates the important role of light signaling pathways in plant resistance to nematodes, providing a new perspective for RKN resistance research.

**Supplementary Information:**

The online version contains supplementary material available at 10.1186/s12870-024-05356-2.

## Background

Outbreaks of plant-parasitic nematodes can cause severe economic losses, estimated at $77 - $157 billion each year [1]. Root-knot nematodes (RKNs, *Meloidogyne* spp.) are among the most widespread and damaging plant-parasitic nematodes worldwide. They can infect more than 5500 hosts, including crops, vegetables and fruits [2]. Three species of “tropical” root knot nematodes, *Meloidogyne incognita*, *Meloidogyne javanica* and *Meloidogyne arenaria*, are among the most rapidly spreading crop pests and pathogens [3]. One of the most important species is *M. incognita*, which causes damage to the global agricultural economy [4].

Light affects seed germination, circadian rhythms, flowering development and shade avoidance. These responses are mediated by several classes of photoreceptors, which absorb light of different wavelengths [5]. The main photoreceptors include phytochrome, which absorbs red and far-red light, and cryptochrome and phototropin, which absorb blue light. Photoreceptors play important roles in the interaction between the host and pathogen [6]. Compared with exposure to white light, cucumber exposure to red light resulted in higher levels of H_2_O_2_ and salicylic acid (SA) and stronger expression of defense genes such as *PR-1*. Therefore, red light enhances salicylic acid pathway-mediated resistance in cucumber [7]. In addition, red light treatment improves tomato resistance to *Pseudomonas syringae* [8]. Under blue light, *CRY2/PHOT2* negatively regulates the proteasome-mediated degradation of HRT, likely via *COP1*, and blue light relieves this repression, resulting in HRT degradation, making plants resistant to *turnip crinkle virus* (TCV) [9]. The blue photoreceptor *CRY1* has been identified in plants. The expression of the salicylic acid (SA)-induced pathogenesis-related gene *PR-1* is reduced in *cry1* mutants but is enhanced in *cry1-ox* plants. Therefore, the blue light photoreceptor *CRY1* positively regulates inducible resistance to *P. syringae* [10].

Photoreceptors activate many intermediary transcription factors after absorbing different wavelengths, and several transcription factors that act downstream of either single or multiple photoreceptors have been functionally characterized [11, 12]. Among them, ELONGATED HYPOCOTYL5 (*HY5*) is a member of the basic-region leucine zipper (bZIP) family of transcription factors [13] and is involved in photomorphogenesis downstream of phytochromes, cryptochromes and photoreceptors [14]. In recent years, the functions of *HY5* have been linked to the plant defense response signaling pathway. *HY5/HYH* directly bind to the promoters of the reactive oxygen species (ROS) signal-related genes *APX2, ZAT10, SIB1, ERF4* and *NDB2* [15]. Enhanced disease susceptibility 1 (*EDS1*) is a positive regulator of the basal resistance of plants to biological stress. *HY5* enhances *EDS1* expression by binding to the G-box of the *EDS1* promoter and regulating the defense response in plants [16]. The plant hormone auxin (IAA) regulates plant disease responses. *HY5* binds to the promoters of *SLR* and *AXR2*, which are negative regulators of auxin signaling, thereby regulating plant resistance to pathogens [17]. Brassinosteroids (BRs) play important roles in plant stress resistance, and *HY5/HYH* can directly bind to the promoter region of *MSBP1* to inhibit BR synthesis [18]. *HY5* affects both sucrose metabolism and shoot‒root transport by promoting the expression of *TPS1*, a gene encoding trehalose-6-phosphate synthase, and *SWEET11* and *SWEET12*, genes encoding sucrose efflux transporters [19].

Light signaling plays an important role in pathogen infection and development. *HY5*, a core transcription factor involved in light signaling, has been extensively studied with respect to plant physiology. However, its involvement in the infection and development of plant-parasitic nematodes has not been reported. In this study, we revealed the mechanism through which photoreceptors, *HY5*, and *SWEETs* jointly influence RKN infection and development.

## Methods

### Plant material and growth conditions

Seeds for the *phya-211, phyb-9, phya/b, cry1/2, phot1/2, cry1/2 phot1/2, hy5, pHY5:HY5-GFP hy5, sweet11a, sweet12b, sweet15d* and *sweet11a12b15d* lines were obtained from the Carnegie Institution for Science. All the mutants were generated in the Col-0 background. Col-0 was used as the wild-type control. Seeds were vernalized at 4 °C for 72 h and germinated in plastic Ray Leach containers (4 cm in diameter and 13.5 cm high) containing equal ratios of sterilized sand and potting substrate (pH: 6.5–6.8; N, P, K ≥ 12 g/kg; water content ≤ 40%; organic content ≥ 40%; Si ≥ 0.3 g/kg) under the conditions of a 16-h/8-h photophase, 23–26 °C, and 50% relative humidity.

For light treatment, *Arabidopsis* (Col-0) seedlings were grown under white light (400–720 nm, 200 µmol m^− 2^s^− 1^) conditions (16 h light/8 h dark) or in continuous darkness. For the various wavelength experiments, after germination, *Arabidopsis* seedlings were grown under blue light (400–500 nm, 200 µmol m^− 2^s^− 1^) or red light (620–720 nm, 200 µmol m^− 2^s^− 1^). After germination, 10-day-old seedlings were inoculated with second-stage juveniles. Fifteen plants were used for each biological replicate.

### Second-stage juvenile collection and inoculation

*Meloidogyne incognita* worms were maintained on a nematode-susceptible tobacco cultivar (Honghuadajinyuan) at the Nematode Institute of Northeastern China. Eggs were collected as described previously [20], with modifications. The roots were cut into pieces and then shaken with 10% commercial bleach for 5 min. The roots were poured through an 80-mesh sieve (180 μm), and the eggs were collected on a 500-mesh sieve (25 μm). The eggs were quickly purified by centrifugation in 35% sucrose for 10 min. The mixture was subjected to another round of centrifugation for 5 min and then rinsed three times in sterile water. The eggs were transferred to a modified Baermann pan at 25 °C in the dark, and the freshly hatched preparasitic second-stage RKN juveniles (J2s) were then harvested.

For RKN infection and development assays, ten days after the *Arabidopsis* seedlings geminated, three holes were dug around the *Arabidopsis* roots. One milliliter of 0.1% water‒agar mixture containing approximately 1000 J2s or water‒agar mixture alone was used as a treatment or control, respectively. At 18 days after inoculation, the seedlings were removed from the container for staining. The roots of the plants were treated with 10% bleach for 1 min, washed well with water, and boiled for 1 min in acid fuchsin solution (3.5% acid fuchsin in 25% acetic acid). After the solution cooled to room temperature, the acid fuchsin solution was washed away with water. The root material was then placed in 30 ml of glycerin acidified with a few drops of 6 N CH_3_COOH and heated to boiling [21]. Fifteen plants were used for each biological replicate. The number of galls and nematodes was counted using a Nikon SMZ800 stereomicroscope (Nikon, Japan). Nematode development was calculated by the following equation: juvenile nematode number/total number of nematodes. The standards for counting nematodes at different developmental stages are shown in Figure S2.

### RNA extraction and quantitative PCR

*Arabidopsis* roots and leaves were harvested on Day 1 and Day 15 post-RKN inoculation (dpi) for gene expression investigation. Three independent biological replicates and three controls (plants at the same stage but without RKN inoculation) were used for RNA extraction at each time point. Total RNA was extracted using TRIzol reagent (Dingguo Biotechnology Co., Ltd., China). cDNA was synthesized from the extracted RNA samples using the PrimeScript RT reagent kit (Takara Bio, Japan) according to the manufacturer’s protocol.

Quantitative PCRs were performed in 96-well hard-shell PCR plates using a One Step SYBR PrimeScript RT‒PCR kit (Takara Bio, Tokyo, Japan) in a 20 µl volume. The reaction conditions were as follows: 95 °C for 30 s, 40 cycles at 95 °C for 5 s, and 60 °C for 30 s. Gene expression was calculated using the 2^−ΔΔCt^ method. To ensure accurate qPCR results, the expression stability under nematode infection conditions of four candidate reference genes (*AtACTIN1*, *AtACTIN8, AtOXA1* and *AtUBP22*) was evaluated using geNorm, NormFinder and BestKeeper analyses. The *ATACTIN8* gene was used as an internal control for normalization of gene expression. Three technical replications were applied for each sample. All the primers used for quantitative PCR are shown in Table S1. T tests were utilized for measuring the significance of differences in gene expression, and *p* values were corrected with a false discovery rate (FDR).

### Chromatin immunoprecipitation (ChIP) assays

Chromatin immunoprecipitation (ChIP) was performed as described previously [22], with slight modifications. Ten-day-old roots of HY5-GFP seedlings in the *hy5* background were harvested. In brief, the samples were crosslinked using 1% formaldehyde for 15 min under a vacuum, and then 2 M glycine was added for an additional 15 min to terminate crosslinking. The samples were washed three times with ddH_2_O, frozen in liquid nitrogen and ground into a fine powder. We produced chromatin fragments (300∼500 bp) through sonication with a bioruptor (Bioruptor Plus; program 30 s on and 30 s off for 3 min). Anti-GFP-coupled Dynabeads were used for immunoprecipitation. After washing with low-salt wash buffer, high-salt wash buffer, LiCl wash buffer, and TE buffer, the samples were eluted with elution buffer. After the addition of 5 M NaCl, the samples were incubated overnight at 65 °C for reverse crosslinking. Immunoprecipitates were analyzed by semiquantitative PCR. Each input DNA level was used as an internal control. The PCR primers used for the ChIP assay are listed in Table S1.

### Yeast one-hybrid analysis

The 386 bp *AtSWEET11*, 369 bp *AtSWEET12*, and 284 bp *AtSWEET15* promoters were cloned and inserted into the *pAbAi* (Takara Bio, Japan) vector, and the open reading frame (ORF) sequence of *AtHY5* was cloned and inserted into the *pGAD7* (Takara Bio, Japan) vector. The appropriate *pAbAi-AtSWEETs* plasmid was transformed into the yeast strain Y1H Gold. Transformants containing each *AtSWEETs* promoter were used as competent cells and transformed with *pGAD7-AtHY5* or a *pGAD7* empty vector. The growth of yeast cells on -Leu synthetic dropout media was monitored.

### Statistical analysis

All the data were analyzed using SPSS Statistics v.22.0. and GraphPad Prism 8 software. One-way analysis of variance (ANOVA) was conducted in this study. For ANOVAs, the data were tested for normality and equality of variations, and if necessary, natural log transformations were performed. All the data were analyzed using paired t tests and Tukey’s multiple comparison tests. The observed differences were found to be statistically significant (*p* < 0.05).

## Results

### Light is required for nematode infection and development

**Fig. 1:**
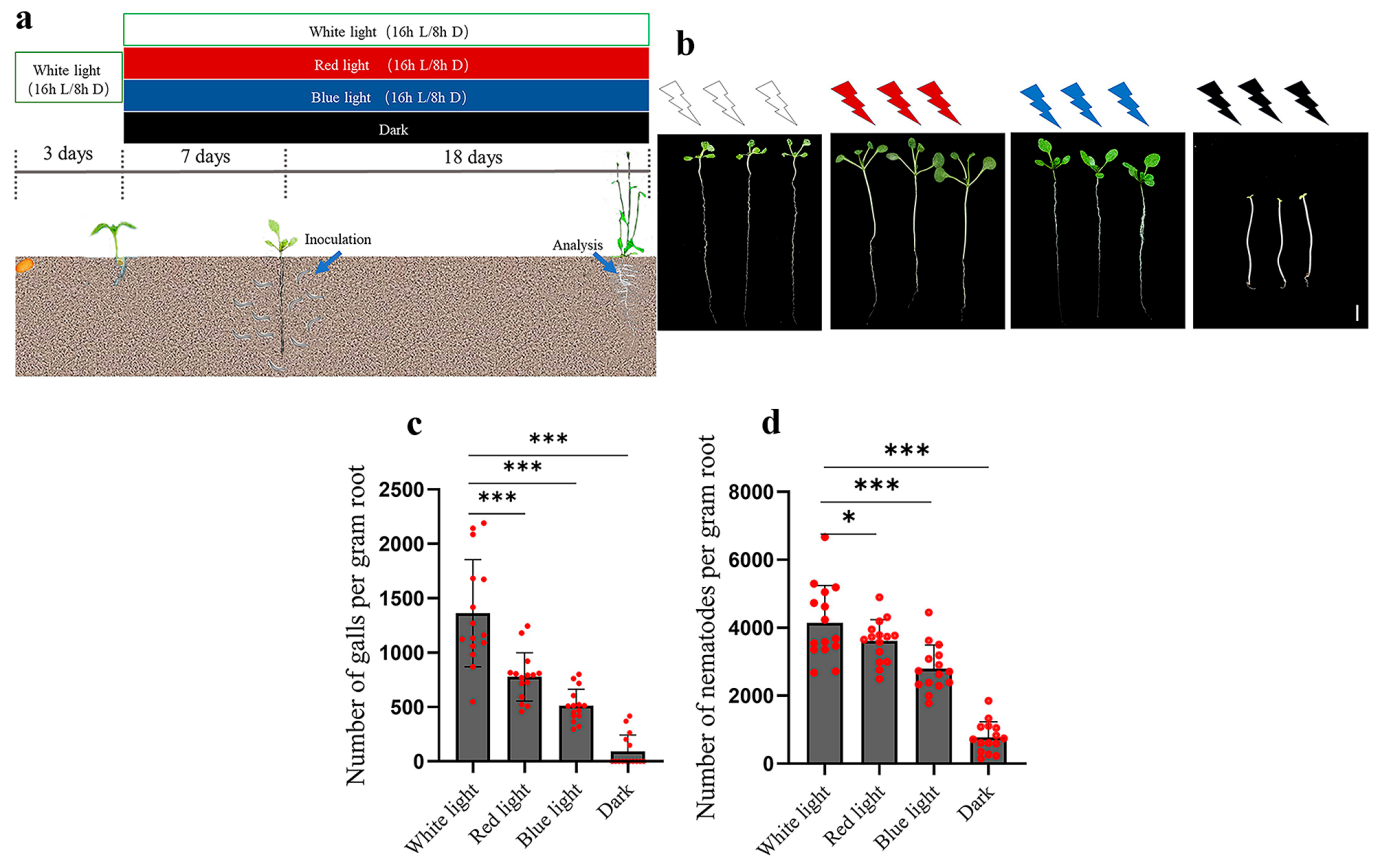
(**a**) Schematic diagram of the nematode infection and development assays under different light conditions. (**b**) Growth phenotype of *Arabidopsis thaliana* under different light conditions at 10 dpi. Scale bar, 5 mm. (**c**) (**d**) Effects of light on *M. incognita* infection of Col-0 at 18 dpi, as determined by acid fuchsin solution staining. The data are the means ± SDs (*n* = 15). (*P < 0.05; **P < 0.01; ***P < 0.001, two-tailed t test)

Throughout the plant’s life cycle, light, as an environmental signal, influences plant growth. To explore the influence of different wavelengths of light on nematode infection and development, we pretreated germinating *Arabidopsis* (Col-0) seedlings, which reached the soil surface under different wavelengths of light, to induce plant light responses to the different wavelengths, and inoculated them with RKN (Fig. 1a). The *Arabidopsis* seedlings demonstrated diverse development phases when exposed to the different wavelengths of light for 10 days (Fig. 1b). To explore the role of light in nematode infection and development, 10-day-old *Arabidopsis* seedlings were subjected to RKN penetration assays under different light conditions. The number of galls and nematodes significantly decreased in the dark. We therefore assessed these processes using red and blue light. There were more galls and nematodes under red light than under blue light, and the numbers of galls and nematodes under all light treatments were significantly greater than those under continuous darkness (Fig. 1c, d). Thus, RKN infection is affected by light signals.

### Response of the red light photoreceptor phytochrome to RKN parasitism in Arabidopsis

**Fig. 2:**
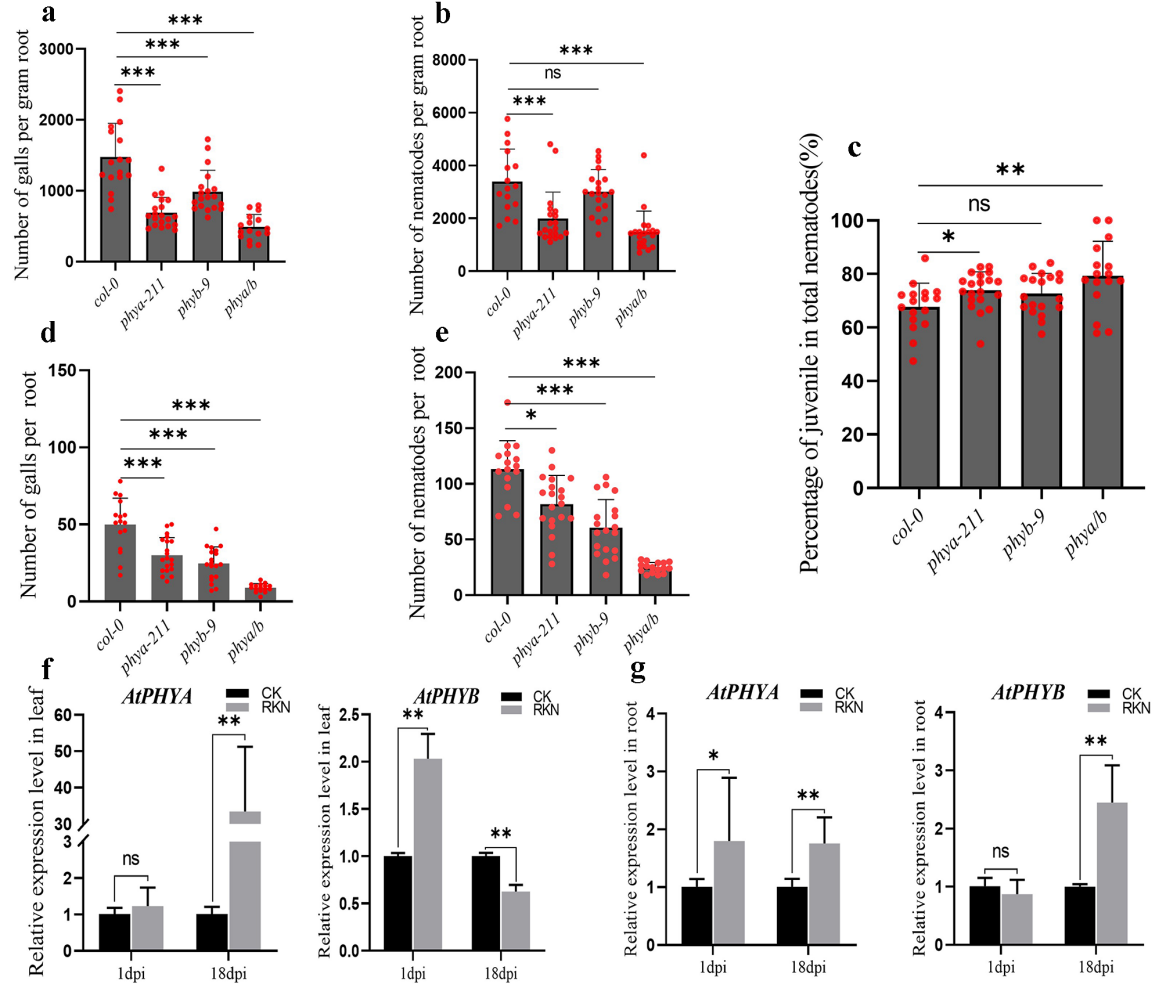
(**a**) (**b**) (**c**) The fresh weight of each root was measured 18 days after inoculation of *phya-211, phyb-9* or *phya/b*. The number of galls infected by nematodes and the ratio of juveniles to total nematodes in each root system per gram of root were analyzed. (**d**) (**e**) The number of galls and nematodes in each root system was analyzed. The data are the means ± SDs (*n* = 15). (**f**) (**g**) qPCR was used to quantify *AtPHYA* and *AtPHYB* expression at 1 dpi and 18 dpi in *Arabidopsis*. Three technical repeats were performed per sample. The *Actin8* gene was used as an internal control. The error bars indicate the SDs of technical repeats (*n* = 3). (*P < 0.05; **P < 0.01; ***P < 0.001, two-tailed t test)

The *Arabidopsis* genome encodes five red light photoreceptor phytochromes (phyA–phyE) [23]. Among these genes, phyA and phyB are the most commonly reported to be associated with plant resistance [24, 25]. *Col-0, phya-211, phyb-9*, and *phya/b* seedlings were grown vertically on 1/2 MS medium under white light, and the observed root lengths of *phya-211, phyb-9*, and *phya/b* were significantly shorter than those of *Col-0* (Figure S1). The number of galls was calculated at 18 days after inoculation, and significantly lower numbers of galls were observed on *phya-211, phyb-9* and *phya/b* mutant roots than on wild-type roots (Fig. 2a). The total number of nematodes was lower in the roots of *phya-211* and *phya/b* mutants than in those of the wild type. However, no significant difference was observed between the wild type and the *phyb* mutant (Fig. 2b). Compared with those in the wild-type control, juveniles were present in greater proportions in the *phya-211* and *phya/b* mutants, but no differences were found in the *phyb* mutant (Fig. 2c). Compared with the nonnormalized phenotypic results, the numbers of galls and nematodes were generally higher in the *phya-211* and *phyb-9* mutants, possibly because the root weights of the *phya-211* and *phyb-9* mutants were much lower than that of Col-0 (Fig. 2d, e). At the same time, wild-type *Arabidopsis* seedlings were inoculated with J2 *M. incognita*, and qPCR analysis of the expression of the *PHYA/PHYB* gene was performed during the infection (1 dpi) and development (18 dpi) periods. In *Arabidopsis* leaves, *PHYA* exhibited the greatest increase (approximately 30-fold) at 18 dpi in response to RKN infection (Fig. 2f). In *Arabidopsis* roots, *PHYA* was induced at 1 dpi by RKN infection, and *PHYA* and *PHYB* were induced at 18 dpi (Fig. 2g).

### Blue light photoreceptors cryptochrome and phototropin in the *Arabidopsis* response to RKN parasitism

**Fig. 3:**
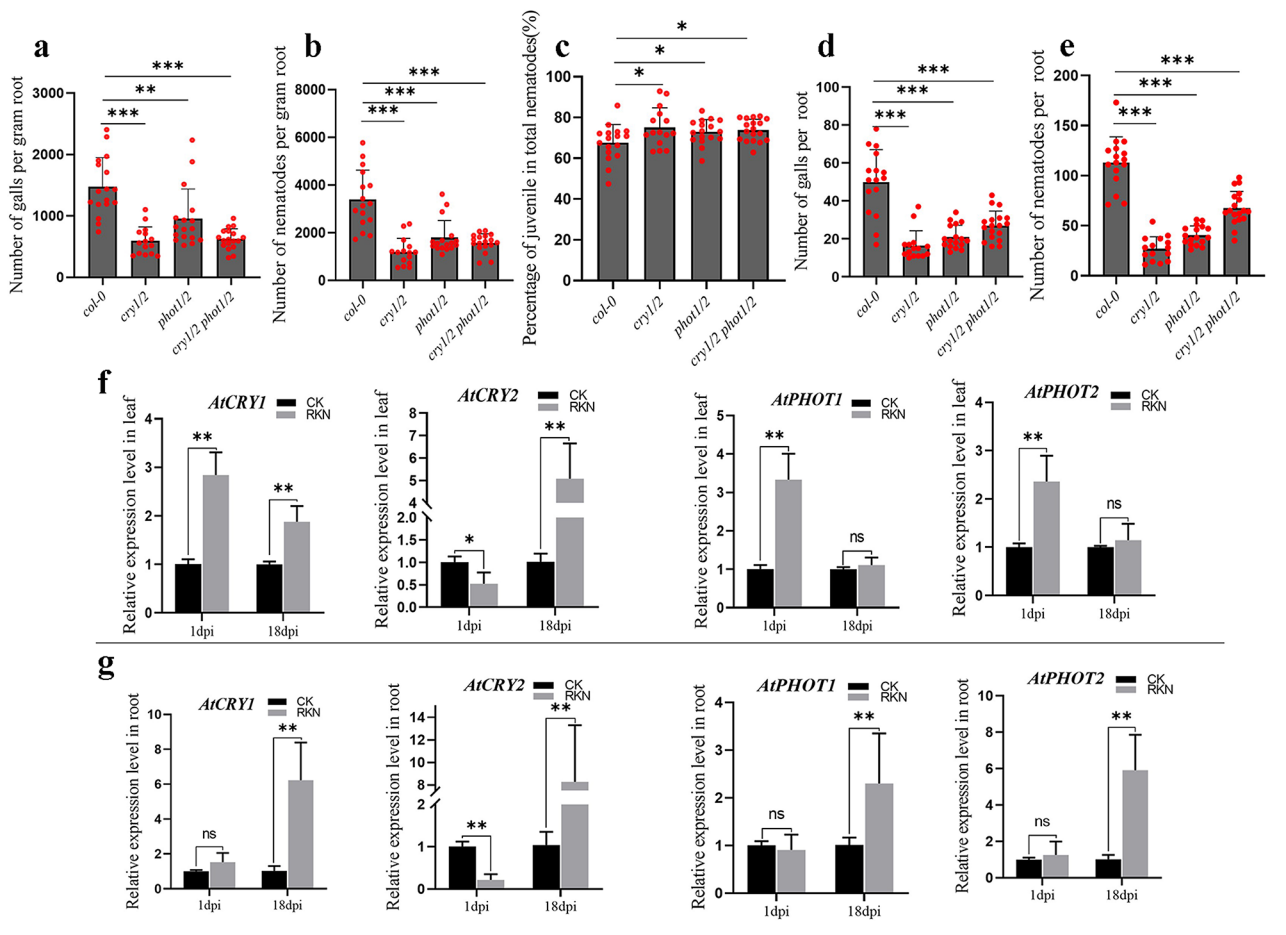
(**a**) (**b**) (**c**) The fresh weight of each root was measured 18 days after inoculation of *cry1/2, phot1/2* and *cry1/2 phot1/2.* The number of galls infected by nematodes and the ratio of juveniles to total nematodes in each root system per gram of root were analyzed. The data are the means ± SDs (*n* = 15). (**d**) (**e**) The number of galls and nematodes in each root system was analyzed. (**f**)(**g**) qPCR was used to quantify *AtCRY1, AtCRY2, AtPHOT1*, and *AtPHOT2* expression at 1 dpi and 18 dpi in *Arabidopsis*. Three technical repeats were performed per sample. The *Actin8* gene was used as an internal control. The error bars indicate the SDs of technical repeats (*n* = 3). (*P < 0.05; **P < 0.01; ***P < 0.001, two-tailed t test)

Arabidopsis has two kinds of blue light photoreceptors, cryptochromes and phototropins [26]. *cry1/2, phot1/2, and cry1/2 phot1/2* seedlings (mutants of the two types of blue light photoreceptors) were grown vertically on 1/2 MS medium under white light, and the lengths of the roots of *cry1/2, phot1/2, and cry1/2 phot1/2* were significantly shorter than those of *Col-0* (Figure S1). The *cry1/2, phot1/2*, and *cry1/2 phot1/2* mutants and the wild type were used to analyze the effect of blue light signaling on RKN invasion and development. The number of galls was calculated at 18 days after J2 inoculation, and fewer galls were observed in the *cry1/2, phot1/2, and cry1/2 phot1/2* mutants than in the wild type (Fig. 3a). Additionally, fewer total nematodes were detected in the *cry1/2, phot1/2*, and *cry1/2 phot1/2* mutants than in the wild-type plants (Fig. 3b). Juveniles were present in greater proportions in *cry1/2, phot1/2*, and *cry1/2 phot1/2* mutant roots (Fig. 3c). The normalized and nonnormalized phenotype results were the same for the blue light photoreceptor mutants (Fig. 3d, e). To further confirm the induction of *CRY* and *PHOT* by RKN, the expression of these genes was analyzed by qPCR during the infection (1 dpi) and development (18 dpi) periods. In *Arabidopsis* leaves, the expression of *CRY1, PHOT1* and *PHOT2* was significantly induced by RKN infection at 1 dpi compared with that in the control group. *CRY1* and *CRY2* were induced at 18 dpi compared with the control group (Fig. 3f). In *Arabidopsis* roots, *CRY1, CRY2, PHOT1* and *PHOT2* were induced at 18 dpi (Fig. 3g).

### Photoreceptor-mediated RKN resistance through HY5

**Fig. 4:**
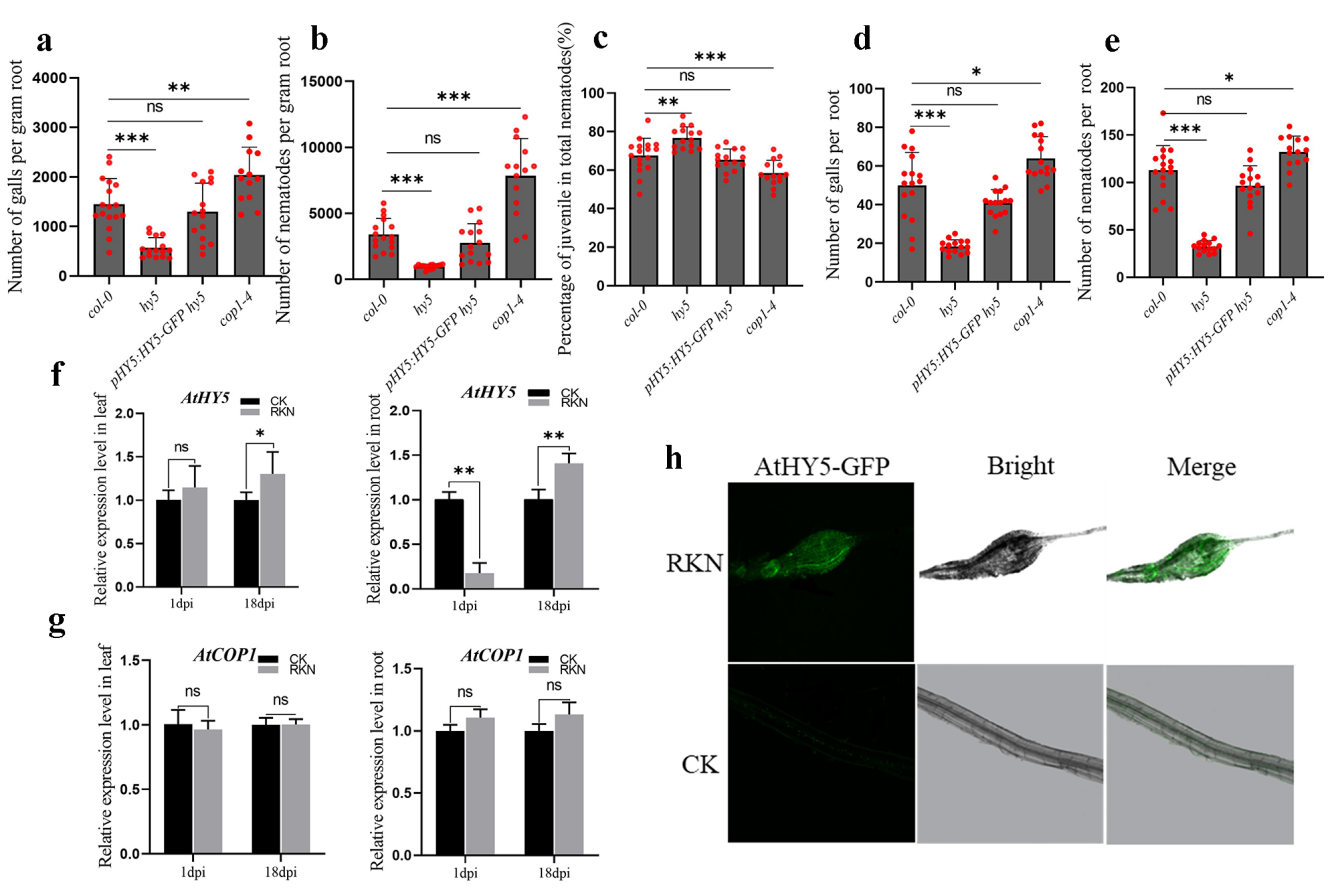
(**a**) (**b**) (**c**) The fresh weight of each root was measured 18 days after inoculation of *hy5, pHY5:HY5-GFP hy5* and *cop1-4.* The number of galls infected by nematodes and the ratio of juveniles to total nematodes in each root system per gram of root were analyzed. The data are the means ± SDs (*n* = 15). (**d**)(**e**) The number of galls and nematodes in each root system was analyzed. (**f**) (**g**) qPCR was used to quantify *AtHY5* and *AtCOP1* expression at 1 dpi and 18 dpi in *Arabidopsis*. Three technical repeats were performed per sample. The *Actin8* gene was used as an internal control. The error bars indicate the SDs of technical repeats (*n* = 3). (*P < 0.05; **P < 0.01; ***P < 0.001, two-tailed t test). (**h**) Intensity of plants inoculated with the green fluorescent protein AtHY5-GFP and *Meloidogyne incognita* were measured by laser scanning confocal microscopy

Photoreceptors sense and transduce light information to downstream signaling pathways. The transcription factor *HY5* is located downstream of photoreceptors and plays an important role in the light signaling pathway. *COP1* is a central negative regulator of photomorphogenesis that physically interacts with *HY5* [27]. HY5 interacts with *COP1* to specifically target *HY5* for proteasome-mediated degradation in the nucleus [28]. *hy5* and *cop1-4* seedlings were grown vertically on 1/2 MS medium with a 12 h light photoperiod, and the root lengths of *hy5* and *cop1-4* were significantly shorter than that of *Col-0* (Figure S1). The susceptibility of the *hy5, pHY5:HY5-GFP hy5*, and *cop1-4* lines and the wild type to RKN infection was studied. The number of galls and nematodes in *pHY5:HY5-GFP hy5* was not significantly different from that in the control group. However, in contrast to those in the wild type treatment, the number of galls and nematodes in *hy5* was significantly lower, and the opposite phenotype was observed in *cop1-4* (Fig. 4a, b). There was a greater proportion of juveniles in the roots of the *hy5* mutant and a lower proportion of juveniles in the roots of the *cop1-4* mutant than in the wild-type roots at 18 dpi. (Fig. 4c). The normalized and nonnormalized phenotype results were the same for the *hy5* and *cop1-4* mutants (Fig. 4d, e). The relative expression levels of the *HY5* gene were determined during infection (1 dpi) and development (18 dpi). Compared with that in the control group, the expression of *HY5* was significantly higher in the *Arabidopsis* leaves at 18 dpi (Fig. 4f). In the *Arabidopsis* roots, *HY5* expression was suppressed at 1 dpi and induced at 18 dpi compared with that in the control group (Fig. 4f). However, *COP1* expression was not induced after infection (Fig. 4g), indicating that *HY5*, rather than *COP1*, was the main nematode target gene. To further analyze whether *HY5* is regulated by RKN infection, fluorescence microscopy was used to detect GFP signals in giant cells. The HY5-GFP signal indicated accumulation in giant cells (Fig. 4h).

### HY5 activates SWEETs by binding to its promoter directly

**Fig. 5:**
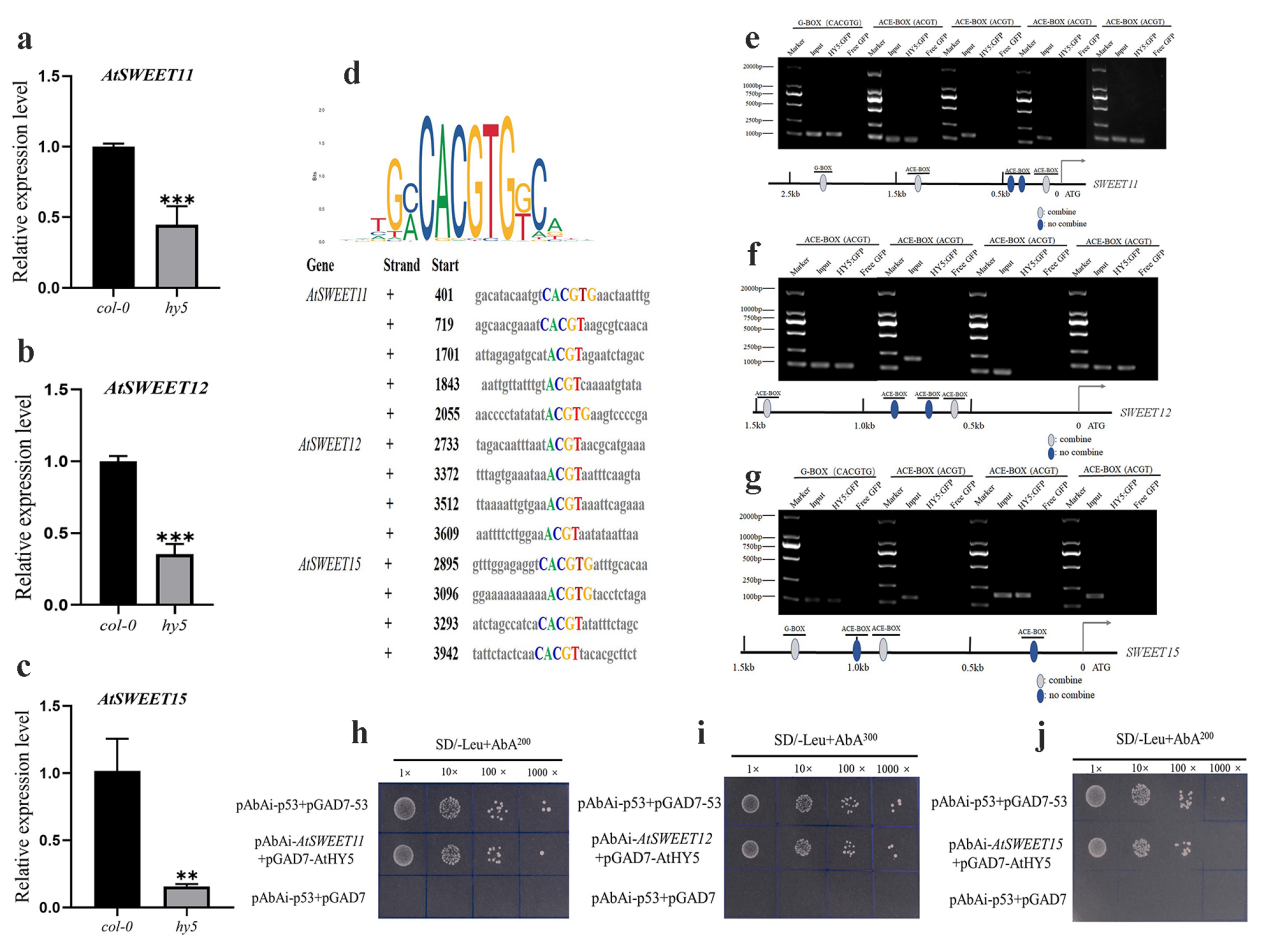
(**a**) (**b**) (**c**) qPCR was used to quantify *AtSWEET11, AtSWEET12*, and *AtSWEET15* expression in *Col-0* and *hy5*. Three technical repeats were performed per sample. The *Actin8* gene was used as an internal control. The error bars indicate the SDs of technical repeats (*n* = 3). (*P < 0.05; **P < 0.01; ***P < 0.001, two-tailed t test). (**d**) Identification of HY5 binding motifs by the JASPAR program. The *SWEET* gene promoters were used for HY5 binding motif analysis. (**e**) (**f**) (**g**) Antiserum against green fluorescent protein (GFP) (Ab) and preimmune serum (Pre) were used for ChIP assays in *Arabidopsis* roots expressing *HY5*:GFP. The areas containing a G-BOX or ACE-BOX were amplified from immunoprecipitated DNA. Input DNA was used as an internal control. (**h**) (**i**) (**j**) A yeast one-hybrid assay was performed to analyze the HY5 activation of the 386 bp *SWEET11*, 369 bp *SWEET12*, and 284 bp *SWEET15* promoters. Yeast cells harboring either pAbAi-p53 + pGAD7-53, pAbAi-*AtSWEETs* + pGAD7-AtHY5 or pAbAi-p53 + pGAD7 were grown on synthetic dropout media lacking Leu (-L)

SWEETs provide bidirectional sugar transfer and are involved in the interaction of numerous pathogenic bacteria and plants. To date, 17 *SWEET* genes have been identified in *Arabidopsi*s [29]. The expression of the clade III sucrose transporters *AtSWEET11, 12* and *15* is significantly increased when plants are challenged with bacterial or fungal pathogens [29]. Infection of *Arabidopsis* by the protist *Plasmodiophora brassicae* led to the phloem-specific accumulation of the *AtSWEET11* and *AtSWEET12* proteins at the site of infection, which facilitated the delivery of sugars to the pathogen [30]. The *atsweet11;12* double-knockout mutants were resistant to the hemibiotrophic fungus *Colletotrichum higginsianum* [31]. Infection with *Botrytis cinerea* enhances the expression of different *AtSWEETs*, principally *AtSWEET15* [29]. *AtSWEET11, 12* and *15* clearly exert their main functions during pathogen infection. *HY5* is a transcription factor involved in light signaling pathways. It is possible that *HY5* mediates the expression of the *SWEET* genes. To investigate this possibility, *SWEET11, SWEET12* and *SWEET15* expression was examined in the *hy5* mutant. qPCR analysis revealed that the expression of the *SWEET11, SWEET12*, and *SWEET15* genes was significantly lower in the *hy5* mutant than in the wild type (Fig. 5a, b,c). As *SWEET11, SWEET12* and *SWEET15* are sensitive to *HY5* expression, it is possible that *HY5* acts as a transcriptional activator of *SWEET11, SWEET12* and *SWEET15.* JASPAR analyses revealed that the *HY5* transcription factor regulates downstream genes by binding to the G-BOX (CACGTG) and ACE-BOX (ACGT) motifs. Moreover, potential binding sites that can be regulated by *HY5* were identified in the promoters of the *SWEET11, SWEET12*, and *SWEET15* genes (Fig. 5d). To obtain evidence that *HY5* binds directly to the *SWEET11, SWEET12*, and *SWEET15* promoters, a ChIP assay was performed using promoter regions from the *SWEET11, SWEET12*, and *SWEET15* loci and a *HY5-GFP* transgenic line. Root tissue from *HY5-GFP* lines was utilized for the ChIP assay. Fragments of approximately 40–140 bp from each part were amplified from immunoprecipitates isolated with anti-GFP antibodies. The *SWEET11* promoter region was divided into five regions, and three regions located between bp 107 and 167, bp 1445 and 1470, and bp 1730 and 1787 from the translation start codon were amplified from the chromatin immunoprecipitates (Fig. 5e). The *SWEET12* promoter region was divided into four regions, and two regions located between bp 577 and 620 and between bp 1454 and 1502 from the translation start codon were amplified from the chromatin immunoprecipitates (Fig. 5f). The *SWEET15* promoter region was divided into four regions, and two regions located between bp 824 and 956 and between bp 1233 and 1289 from the translation start codon were amplified from the chromatin immunoprecipitates (Fig. 5g). ChIP indicated the presence of a *HY5 cis*-regulatory element in a fragment of the *SWEET* promoter. The results of this binding assay were confirmed using a yeast one-hybrid assay, which indicated that *HY5* can activate the *SWEET11, SWEET12* and *SWEET15* promoters (Fig. 5h, i, j). Thus, the G-BOX and ACE-BOX are responsible for the binding of *HY5*. In summary, these data demonstrate that *HY5* activates the transcription of *SWEET11, SWEET12* and *SWEET15* by binding the G-BOX and ACE-BOX *cis*-elements.

### SWEETs negatively regulate RKN resistance

**Fig. 6:**
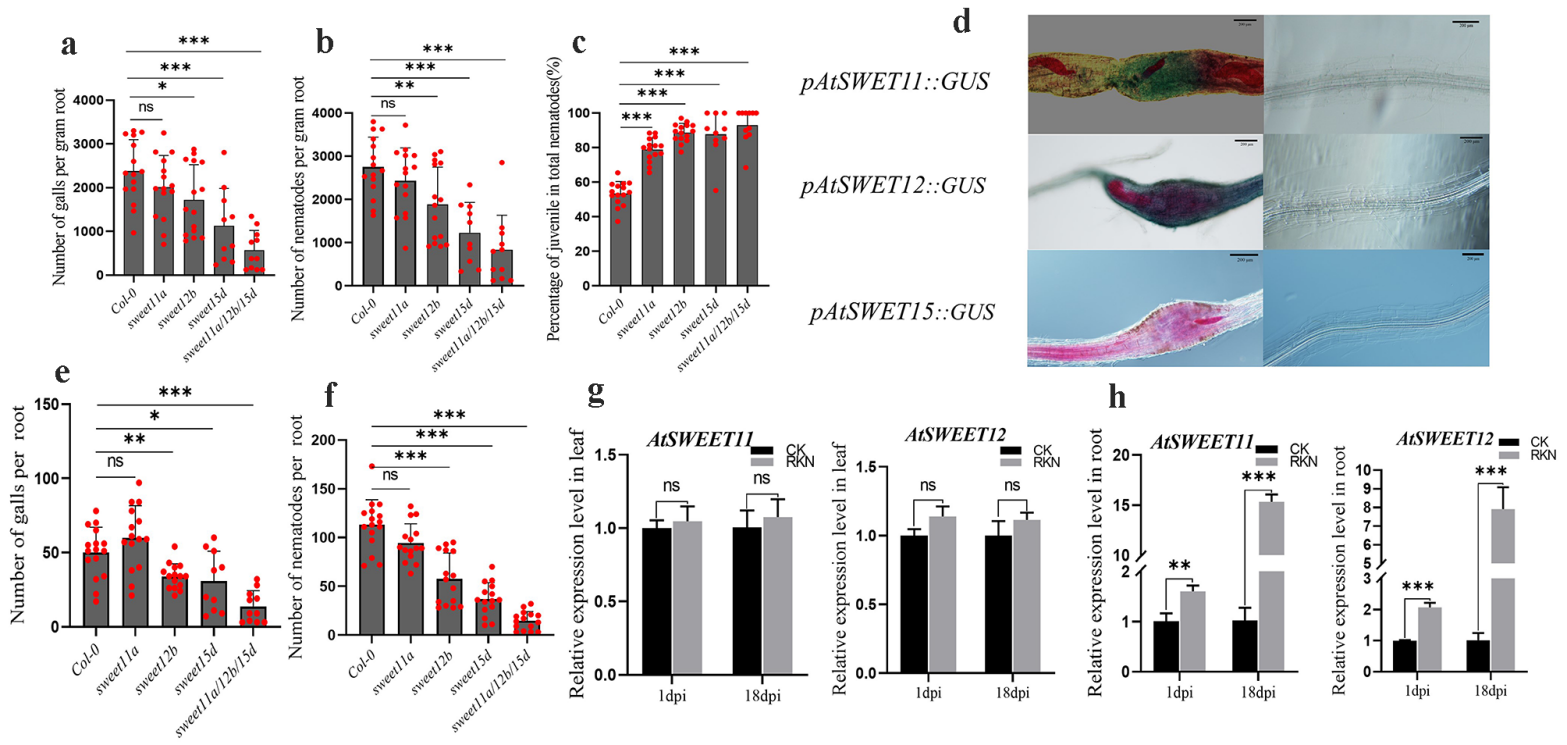
(**a**) (**b**) (**c**) The fresh weight of each root was measured 18 days after inoculation of the *sweet11a, sweet12b, sweet15d* and *sweet11a/12b/15d* mutants. The number of galls infected by nematodes and the ratio of juveniles to total nematodes in the roots were analyzed. The data are the means ± SDs. (**d**) Histochemical GUS assay of AtSWEET11-GUS, AtSWEET12-GUS and AtSWEET15-GUS lines infected with J2s of *M. incognita*. Strong GUS activity at the nematode feeding sites was observed. (**e**)(**f**) The number of galls and nematodes in each root system was analyzed. (**g**) (**h**) qPCR was used to quantify *AtSWEET11* and *AtSWEET12* expression at 1 dpi and 18 dpi in *Arabidopsis thaliana*. Three technical repeats were performed per sample. The *Actin8* gene was used as an internal control. The error bars indicate the SDs of technical repeats (*n* = 3). (*P < 0.05; **P < 0.01; ***P < 0.001, two-tailed t test)

The responses of *sweet11a, sweet12b*, *sweet15d*, *sweet11a*/*12b*/*15d* and the wild type to RKN infection were investigated. Eighteen days after J2 inoculation, the number of galls was measured, and it was found that the *sweet12b*, *sweet15d*, and *sweet11a*/*sweet12b*/*sweet15d* mutant populations had many fewer galls than did the wild type (Fig. 6a). Additionally, the total number of nematodes was lower in the roots of *sweet12b*, *sweet15d*, and *sweet11a*/*12b*/*15d* than in those of the wild-type control (Fig. 6b). Juveniles were present in greater proportions in *sweet11a, sweet12b*, *sweet15d* and *sweet11a*/*12b*/*15d* mutants (Fig. 6c). To investigate whether *AtSWEET11, AtSWEET12* and *AtSWEET15* specifically accumulated in galls after infection, GUS expression patterns upon RKN infection in *Arabidopsis* plants expressing AtSWEET11-GUS, AtSWEET12-GUS and AtSWEET15-GUS under the control of the endogenous promoter were analyzed. Strong AtSWEET11-GUS and AtSWEET12-GUS signals were observed within the developing knot of *M. incognita* in *Arabidopsis* roots. However, AtSWEET15-GUS was not detected, perhaps because *AtSWEET15* was not expressed in *Arabidopsis* roots (Fig. 6d). The normalized and nonnormalized phenotype results were the same in the *sweet* mutants (Fig. 6e, f). qPCR analysis revealed that the expression of the *AtSWEET11* and *AtSWEET12* genes in *Arabidopsis* roots was significantly induced by RKN infection at 18 dpi compared with that in the control group (Fig. 6g, h).

## Discussion

Despite extensive research on the interaction between nematodes and plants [32–37], limited knowledge exists regarding the regulatory mechanisms employed by nematodes to facilitate nutrient transport from hosts for their own sustenance. Our studies showed that the light signaling pathways and sugar transporters of *Arabidopsis* are regulated by RKN infection and feeding.

Light signaling plays a crucial role in regulating physiological processes during normal growth and development, as well as in plant defense responses. Red light has been found to induce systemic resistance against *Meloidogyne incognita* in watermelon and tomato [38, 39]. Pretreatment of *Arabidopsis* with red light (600–700 nm) induces systemic resistance against *M. incognita* [40]. In this study, we found that the number of galls and nematodes in the continuous darkness treatment group was significantly lower than that in the light treatment groups. To investigate the effects of different wavelengths of light on RKN infection and development, we treated RKN-infected *Arabidopsis* seedlings with red and blue light. The results showed that the number of galls and nematodes under red and blue light conditions was significantly greater than that under continuous darkness. Therefore, different wavelengths of light affect the infection and development of nematodes in *Arabidopsis*. In our experiments on the effects of different light regimes on nematode infection, we found that different light regimes lead to morphological and physiological differences, especially because dark treatment leads to shorter roots, and the absolute nematode number does not reflect whether nematode susceptibility is caused by the immune response or root morphology. To reduce the effect of this morphological difference, we used the number of nematodes normalized to the root mass to analyze susceptibility. Nontargeted plant response analyses, such as transcriptome or metabolome analyses, may be useful. The differences in root morphology and nematode susceptibility warrant further investigation.

Dedicated photoreceptors help plants perceive light signals of different wavelengths [41]. Phytochromes (Phys) detect red/far-red light in the range of 600–750 nm; phytochromes exist in two interconvertible forms, Pr and Pfr, which absorb red and far‐red light, respectively. They are synthesized in an inactive Pr form in the cytosol and translocated to the nucleus after conversion to the Pfr form by the absorption of red light. Photoactivated phytochromes move into the nucleus and modulate the activity of several transcription factors [42]. To evaluate the effect of nematode infection on these photoreceptors, we inoculated phytochrome mutants with nematodes. The number of galls and nematodes in *phya-211* and *phya/b* was significantly lower, and the proportion of juveniles to total nematodes was higher. Moreover, the expression of the *PHY* gene was significantly induced by RKN infection compared with that in the control group. Cryptochromes (*CRYs)* and phototropins (*PHOTs*) detect blue light in the range of 315–400 nm. After perceiving different wavelengths of light, these photoreceptors further transmit signals through a cascade to modulate the expression of multiple genes [42]. In addition, after we inoculated cryptochrome and phototropin mutants with J2 RKNs, the number of galls and nematodes in *cry1/2, phot1/2* and *cry1/2phot1/2* was significantly lower, and the proportion of juveniles to total nematodes was higher. The expression levels of the *CRY* and *PHOT* genes were significantly greater in the RKN-infected group than in the control group. Taken together, our results indicate the importance of photoreceptors in host–nematode interactions.

The bZIP transcription factor *HY5* plays critical roles in light signaling. *HY5* acts as a positive regulator of light signaling. *HY5* was shown to bind to the G-box motif in experiments with fragments of the chalcone synthase gene promoter [43]. When we inoculated the *hy5* and *pHY5:HY5-GFP hy5* mutants with nematodes, we found that the number of galls and nematodes in *hy5* was significantly lower, and the proportion of juveniles to total nematodes was higher; there was no difference in the *pHY5:HY5-GFP hy5* mutant. qPCR analysis of the expression of the *HY5* gene in Col-0 showed that *HY5* was significantly induced in the roots by RKN infection relative to the control group. Fluorescence microscopy was used to detect a GFP signal in giant cells; HY5-GFP was found to be accumulated in giant cells. *HY5* activity is regulated by the CONSTITUTIVELY PHOTOMORPHOGENIC 1 (COP1) and SUPPRESSOR OF PHYA 1 (SPA1) complex, which destabilizes *HY5* via the E3 ligase activity of *COP1* in the nucleus, which causes the ubiquitylation necessary for 26 S proteome-mediated degradation [44, 45]. The present study revealed that the number of galls and nematodes in *cop1-4* increased significantly, and the proportion of juveniles to total nematodes decreased. The *COP1* gene was induced by RKN infection.

During pathogen infection, pathogens are capable of inducing metabolic and transcriptomic modifications in their hosts. Sugar metabolism and mobilization are greatly affected during infection. The induction of plant *SWEET* transporters by pathogens has been linked to an increased capacity of pathogens to obtain host-derived sugars for nutrition [46]. *AtSWEET11* and *AtSWEET12* localize to the plasma membrane of the phloem, and *AtSWEET11* and *AtSWEET12* mutations restrict intercellular sucrose transport to the interface of adjacent phloem cells to prevent pathogen infections [47]. Plants promote root growth under drought stress by regulating *AtSWEET11* and *AtSWEET12*, enhancing exoplasmic phloem loading at source tissues and unloading at root sink tissues to transport sucrose from shoots to roots [48]. Our results revealed that the number of galls and nematodes were significantly lower in *sweet12b*, *sweet15d*, and *sweet11a*/*sweet12b*/*sweet15d*, and the proportion of juveniles to total nematodes was greater. Nematode infection induces *AtSWEET11* and *AtSWEET12* in root tissues. The expression of *AtSWEET15* and AtSWEET15-GUS was not detected in roots due to the accumulation of *SWEET15* in the epidermal cells of the seed coat; *SWEET15* is unlikely to contribute to the phloem export of sucrose [49]. The mechanism by which *SWEET15* negatively regulates the infection and development of nematodes needs further investigation. Genome-wide ChIP-chip experiments demonstrated that *HY5* regulates the expression of nearly one-third of genes in *Arabidopsis*, and ~ 3000 of these genes are directly controlled by *HY5* binding [50]. *HY5* activates the transcription of *SWEET11, SWEET12* and *SWEET15* by binding the G-BOX and ACE-BOX *cis*-elements, thus affecting nematode infection and development in *Arabidopsis*.

In our experiments in which different mutants were infected with nematodes, we found that *phya-211* and *phyb-9* exhibited meaningful differences between normalized and nonnormalized nematode or gall counts, possibly because the root weights of the *phya-211* and *phyb-9* mutants are much lower than that of Col-0, resulting in a general increase in the normalized mutant phenotype results. The root weight and nematode susceptibility of the mutants warrant further investigation.

## Conclusions

Light is an important living factor for plants and pathogenic organisms. In this study, we showed that the expression of the enzyme ELONGATED HYPOCOTYL5 (HY5) is significantly induced by infection with *Meloidogyne incognita*. *HY5* inhibits hypocotyl growth and lateral root development and has transcriptional activation activity in *Arabidopsis*. Genetic analysis of a *HY5* genome-edited mutant and revertant plants demonstrated that *HY5* negatively regulates plant resistance to RKN. Expression level and genetic analyses revealed that the photoreceptor genes *PHY, CRY*, and *PHOT* have a negative impact on nematode infection. qPCR analysis revealed that *HY5* activates *SWEET11a, SWEET12b* and *SWEET15d*, three SWEET family sugar transporter genes. The *sweet11a*, *sweet12b, sweet15d* and *sweet11a/sweet12b/sweet15d* mutants are less susceptible to RKN than are wild-type plants, suggesting that these three *SWEETs* negatively regulate plant resistance. ChIP and yeast one-hybrid assays revealed that *HY5* directly activates *SWEET11a, SWEET12b* and *SWEET15d* by binding to their promoter fragments. These results indicate that *HY5* activates *SWEET11a, SWEET12b* and *SWEET15d* to negatively regulate plant resistance. Our work on the relationship between *HY5* and sugar transporters should provide useful information for scientists seeking to understand the effects of light signaling regulation on root-knot nematode infection and development.

### Electronic supplementary material

Below is the link to the electronic supplementary material.

Supplementary Material 1

Supplementary Material 2: Table S1. List of PCR primers used in this study.

Supplementary Material 3

Supplementary Material 4

## Data Availability

The datasets used and/or analyzed during the current study are available from the corresponding author on reasonable request.

## Abbreviations

HY5: ELONGATED HYPOCOTYL 5
COP1: CONSTITUTIVELY PHOTOMORPHOGENIC 1
PHYA, B: Phytochrome A, B
CRY1,2: Cryptochrome 1, 2
PHOT1,2: Phototropin 1, 2
RKN: Root-knot nematode
bZIP: Basic-region leucine zipper
ChIP: Chromatin immunoprecipitation
Y1H: Yeast one-hybrid
dpi: Days postinoculation
