# Supplementary material for: Light signaling regulates root-knot nematode infection and development via HY5-SWEET signaling
Source: BMC Plant Biol. 2024 Jul 11;24:664. doi: 10.1186/s12870-024-05356-2 (PMC11238492; doi:10.1186/s12870-024-05356-2)
Supplement: Supplementary file 1 — Supplementary Material 1 [file 12870_2024_5356_MOESM1_ESM.pptx]

## Slide 1
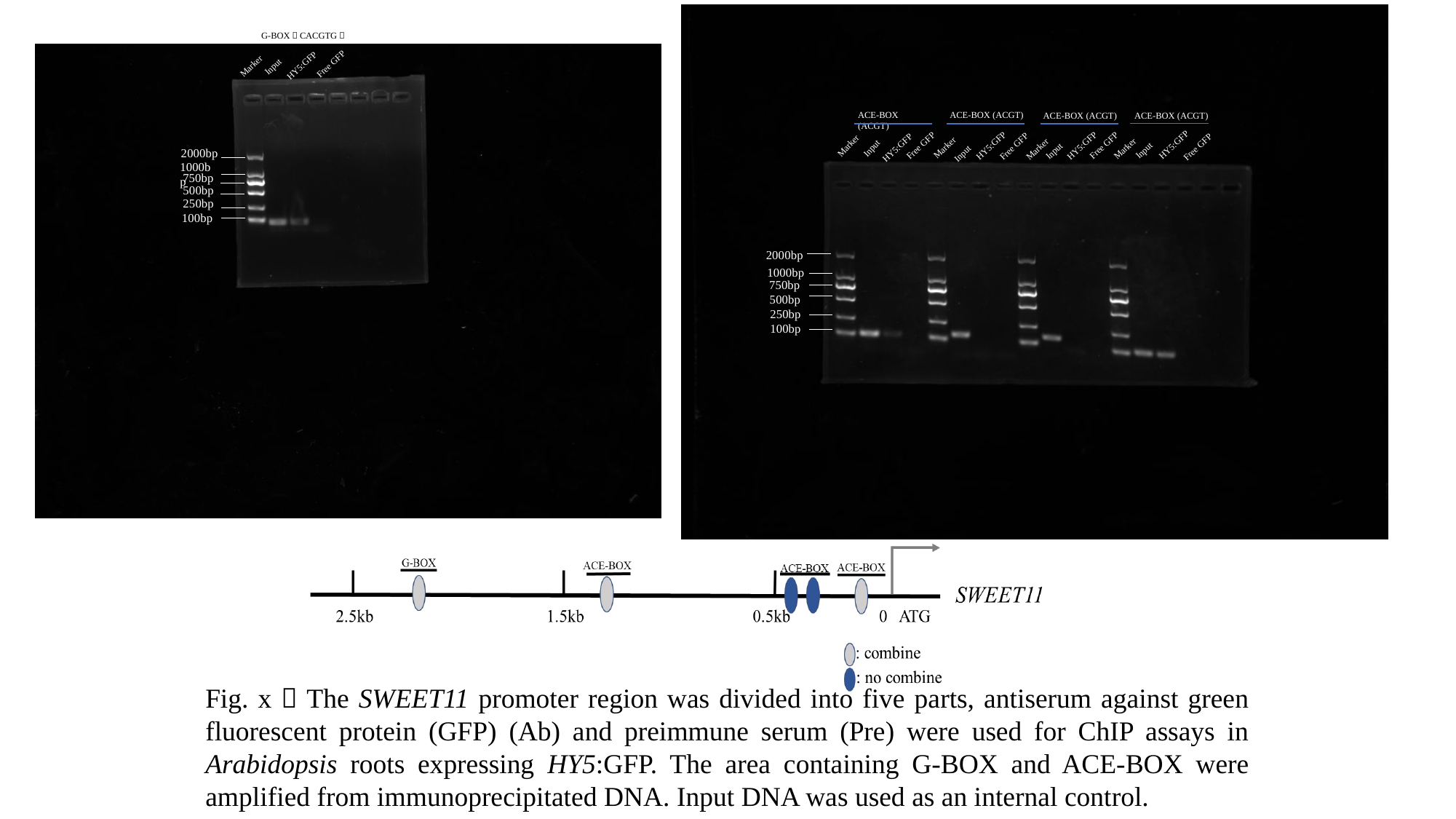

HY5:GFP
G-BOX（CACGTG）
Marker
Input
Free GFP
ACE-BOX (ACGT)
ACE-BOX (ACGT)
ACE-BOX (ACGT)
ACE-BOX (ACGT)
Marker
Marker
Marker
Input
Marker
Input
Input
Input
HY5:GFP
HY5:GFP
HY5:GFP
Free GFP
Free GFP
Free GFP
HY5:GFP
Free GFP
2000bp
1000bp
750bp
500bp
250bp
100bp
2000bp
1000bp
750bp
500bp
250bp
100bp
Fig. x：The SWEET11 promoter region was divided into five parts, antiserum against green fluorescent protein (GFP) (Ab) and preimmune serum (Pre) were used for ChIP assays in Arabidopsis roots expressing HY5:GFP. The area containing G-BOX and ACE-BOX were amplified from immunoprecipitated DNA. Input DNA was used as an internal control.

## Slide 2
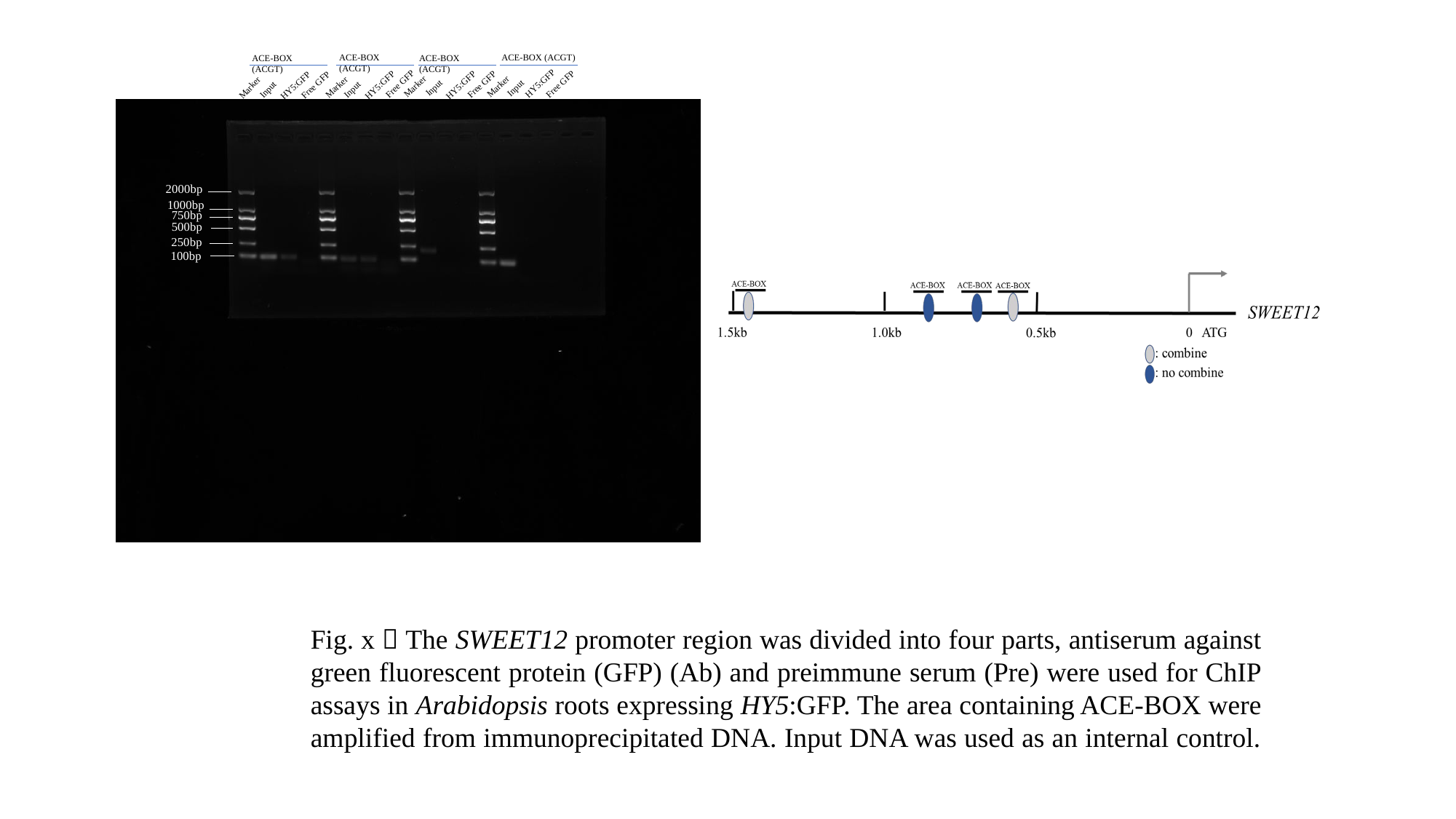

ACE-BOX (ACGT)
ACE-BOX (ACGT)
ACE-BOX (ACGT)
ACE-BOX (ACGT)
Marker
Marker
Marker
Input
Input
Input
Input
HY5:GFP
HY5:GFP
HY5:GFP
Free GFP
Free GFP
Free GFP
HY5:GFP
Free GFP
Marker
2000bp
1000bp
750bp
500bp
250bp
100bp
Fig. x：The SWEET12 promoter region was divided into four parts, antiserum against green fluorescent protein (GFP) (Ab) and preimmune serum (Pre) were used for ChIP assays in Arabidopsis roots expressing HY5:GFP. The area containing ACE-BOX were amplified from immunoprecipitated DNA. Input DNA was used as an internal control.

## Slide 3
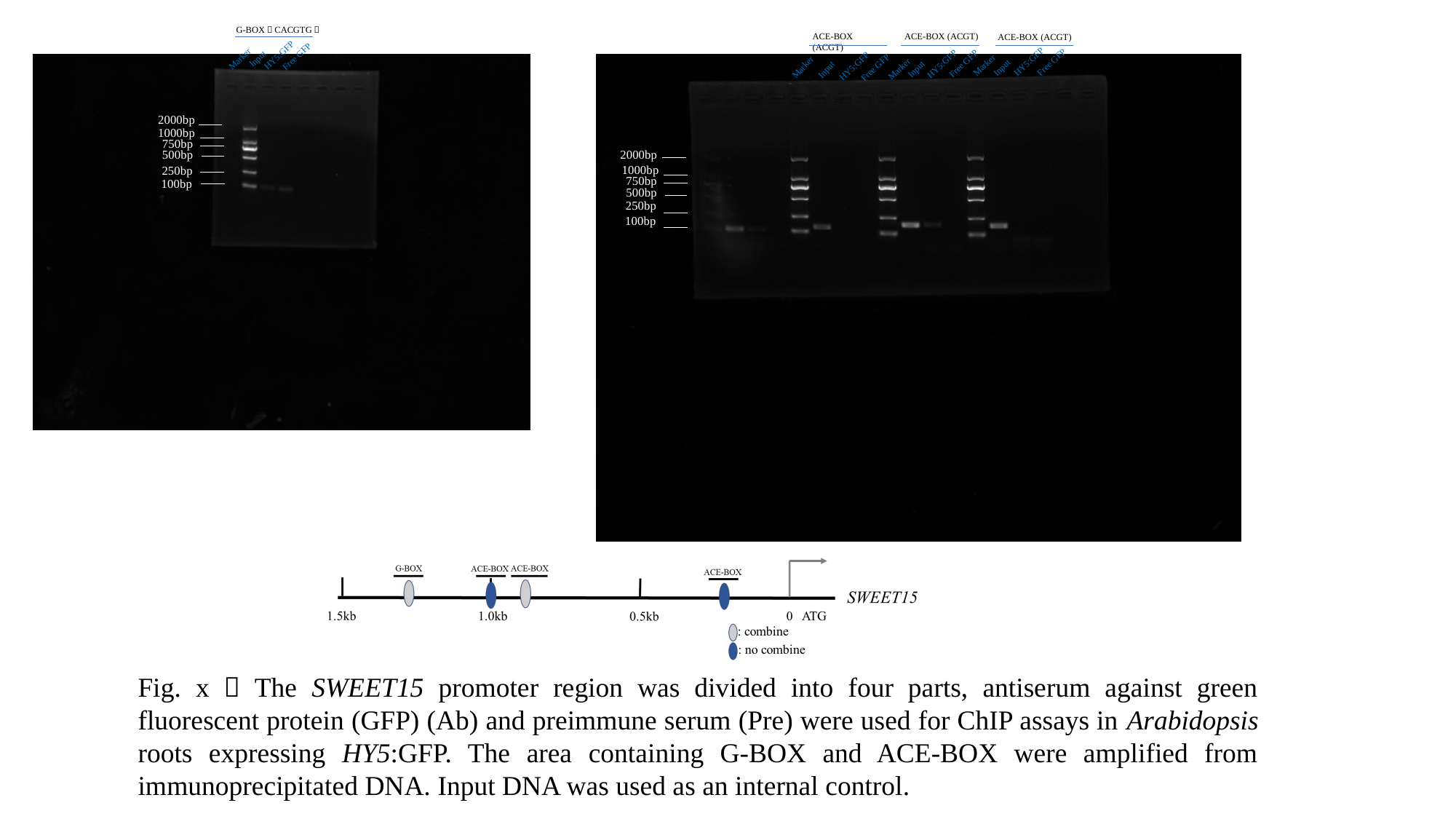

G-BOX（CACGTG）
ACE-BOX (ACGT)
ACE-BOX (ACGT)
ACE-BOX (ACGT)
Marker
Input
Marker
Marker
Marker
Input
Input
Input
HY5:GFP
Free GFP
HY5:GFP
Free GFP
HY5:GFP
Free GFP
HY5:GFP
Free GFP
2000bp
1000bp
750bp
2000bp
500bp
1000bp
250bp
750bp
100bp
500bp
250bp
100bp
Fig. x：The SWEET15 promoter region was divided into four parts, antiserum against green fluorescent protein (GFP) (Ab) and preimmune serum (Pre) were used for ChIP assays in Arabidopsis roots expressing HY5:GFP. The area containing G-BOX and ACE-BOX were amplified from immunoprecipitated DNA. Input DNA was used as an internal control.
