# Supplementary material for: Light signaling regulates root-knot nematode infection and development via HY5-SWEET signaling
Source: BMC Plant Biol. 2024 Jul 11;24:664. doi: 10.1186/s12870-024-05356-2 (PMC11238492; doi:10.1186/s12870-024-05356-2)
Supplement: Supplementary file 2 — Supplementary Material 2: Table S1. List of PCR primers used in this study. [file 12870_2024_5356_MOESM2_ESM.pdf]

The primer of qPCR

Gene	Primer(5'→3')	Sequence
AtPHY-A	*AtPHYA-qPCR-F*	GAAGGATGCTTGGATTTGGA
	AtPHYA-qPCR-R	CCTCAGGGAAGCTGAAACAG
AtPHY-B	*AtPHYB-qPCR-F*	ACATAGCCCAAACCCTCTAATCC
	AtPHYB-qPCR-R	GCGAGACCAACCCGTAAGC
AtCRY-1	*AtCRY1-qPCR-F*	CTCTAAGAACCGCAGAAAAGCC
	AtCRY1-qPCR-R	CTTTTCTCACACTCACTTCCCCA
AtCRY-2	*AtCRY2-qPCR-F*	GCTTTGCTGTGAAGTTTCTTCTCC
	AtCRY2-qPCR-R	GCCTTGTAACGCGGGATTGTC
AtPHOT-1	*AtPHOT1-qPCR-F*	CACTGATCCTAGGCTTCCCG
	AtPHOT1-qPCR-R	GTGGTTAGATCAGTCTCTGGACC
AtPHOT-2	*AtPHOT2-qPCR-F*	GCTACCACTCTTGAGCGCATAGAG
	AtPHOT2-qPCR-R	GCTACCACTCTTGAGCGCATAGAG
AtHY5	*AtHY5-qPCR-F*	CCATCAAGCAGCGAGAGGTCATCAA
	AtHY5-qPCR-R	CGCCGATCCAGATTCTCTACCGGAA
AtCOP1	*AtCOP1-qPCR-F*	TGATGAACTAAATGAGGTGCAAA
	AtCOP1-qPCR-R	TCTGTCCCTAGCTCGGTATAAATC
AtSWEET-11	*AtSWEET11-qPCR-F*	TCCTTCTCCTAACAACTTATATACCAT
	AtSWEET11-qPCR-R	TCCTATAGAACGTTGGCACAGGA
AtSWEET-12	*AtSWEET12-qPCR-F*	AAAGCTGATATCTTTCTTACTACTTCG
	AtSWEET12-qPCR-R	CTTACAAATCCTATAGAACGTTGGCAC
AtSWEET-15	*AtSWEET15-qPCR-F*	CAATGACATATGCATAGCGATTCCAA
	AtSWEET15-qPCR-R	GGACTCATCACGACAATACTCTTAAG
AtACTIN-8	*AtACTIN8-qPCR-F*	CACTTTCCAGCAGATGTGGATC
	AtACTIN8-qPCR-R	AATGCCTGGACCTGCTTCAT

Genes related parameters in qPCR analysis

Gene	Sequence	Amplificon efficiency(%)	Linear correlation coefficient(R^2)
AtPHY-A	GAAGGATGCTTGGATTTGGA<br>CCTCAGGGAAGCTGAAACAG	98.3	0.994
AtPHY-B	ACATAGCCCAAACCCTCTAATCC<br>GCGAGACCAACCCGTAAGC	97.6	0.997
AtCRY-1	CTCTAAGAACCGCAGAAAAGCC<br>CTTTTCTCACACTCACTTCCCCA	95.4	0.998
AtCRY-2	GCTTTGCTGTGAAGTTTCTTCTCC<br>GCCTTGTAACGCGGGATTGTC	100.3	0.988
AtPHOT-1	CACTGATCCTAGGCTTCCCG<br>GTGGTTAGATCAGTCTCTGGACC	97.6	0.996
AtPHOT-2	GCTACCACTCTTGAGCGCATAGAG<br>GCTACCACTCTTGAGCGCATAGAG	94.7	0.997
AtHY5	CCATCAAGCAGCGAGAGGTCATCAA<br>CGCCGATCCAGATTCTCTACCGGAA	93.2	0.994
AtCOP1	TGATGAACTAAATGAGGTGCAAA<br>TCTGTCCCTAGCTCGGTATAAATC	96.7	0.984
AtSWEET-11	TCCTTCTCCTAACAACTTATATACCAT<br>TCCTATAGAACGTTGGCACAGGA	98.4	0.991
AtSWEET-12	AAAGCTGATATCTTTCTTACTACTTCG<br>CTTACAAATCCTATAGAACGTTGGCAC	99.4	0.984
AtSWEET-15	CAATGACATATGCATAGCGATTCCAA<br>GGACTCATCACGACAATACTCTTAAG	97.1	0.987
AtACTIN-8	CACTTTCCAGCAGATGTGGATC<br>AATGCCTGGACCTGCTTCAT	99.9	0.997

The primer of CHIP

Gene	Primer	Sequence
AtSWEET11	*AtSWEET11-ChIP-P1-F*	AGCTAAAGTGAAAACGGCATAAT
	AtSWEET11-ChIP-P1-R	AGAAAGTGAGATTTGCAAAGTGAT
AtSWEET11	*AtSWEET11-ChIP-P2-F*	AGGATTGGTGAAAACCCTCAA
	AtSWEET11-ChIP-P2-R	ATCTCAGCTGTTTAGGCAAGC
AtSWEET11	*AtSWEET11-ChIP-P3-F*	GCTTGCCTAAACAGCTGAGATT
	AtSWEET11-ChIP-P3-R	TCACACGCTAAACCATTACCA
AtSWEET11	*AtSWEET11-ChIP-P4-F*	ACACAAACATTTCTCCTTTTTCCA
	AtSWEET11-ChIP-P4-R	ACGAGCTTAGTGTTTCGGGG
AtSWEET11	*AtSWEET11-ChIP-P5-F*	ACATGTTTCCTTATGTTGACGC
	AtSWEET11-ChIP-P5-R	TCATTTGTAGTTACTTGGTTGGTTT
AtSWEET12	*AtSWEET12-ChIP-P1-F*	AACATTGATCTACTCTTGAAAACGA
	AtSWEET12-ChIP-P1-R	AGGTTGAACCGTCTTTCGTAGT
AtSWEET12	*AtSWEET12-ChIP-P2-F*	TTGCGTACAAACGTCGTCAC
	AtSWEET12-ChIP-P2-R	ACTAAATTACGAGGTCCGTGCT
AtSWEET12	*AtSWEET12-ChIP-P3-F*	TTAGCCGTTTGCCCGTTTCT
	AtSWEET12-ChIP-P3-R	TGTCTATTTCTCTTTCCCTCGGT
AtSWEET12	*AtSWEET12-ChIP-P4-F*	CCGTTTTCACTTTTAGCCACTTTT
	AtSWEET12-ChIP-P4-R	AGAAACGGGCAAACGGCT
AtSWEET15	*AtSWEET15-ChIP-P1-F*	AGTGGAAGCGTTTGGAGAGG
	AtSWEET15-ChIP-P1-R	TGCGTTACGAGAGAGAGTCG
AtSWEET15	*AtSWEET15-ChIP-P2-F*	TTCGACGCGTGGAGTTTTTG
	AtSWEET15-ChIP-P2-R	ACCATTCACATCTCCTGGTCC
AtSWEET15	*AtSWEET15-ChIP-P3-F*	GGGTGGGACTGGTTTCATCT
	AtSWEET15-ChIP-P3-R	AACGGCGTCAATTTTCTGCAA
AtSWEET15	*AtSWEET15-ChIP-P4-F*	TGAGGAGGCTCAGGTGGTTT
	AtSWEET15-ChIP-P4-R	CCACTTGTCATGCAACCAGAC
