# Supplementary material for: Light signaling regulates root-knot nematode infection and development via HY5-SWEET signaling
Source: BMC Plant Biol. 2024 Jul 11;24:664. doi: 10.1186/s12870-024-05356-2 (PMC11238492; doi:10.1186/s12870-024-05356-2)
Supplement: Supplementary file 3 — Supplementary Material 3 [file 12870_2024_5356_MOESM3_ESM.pdf]

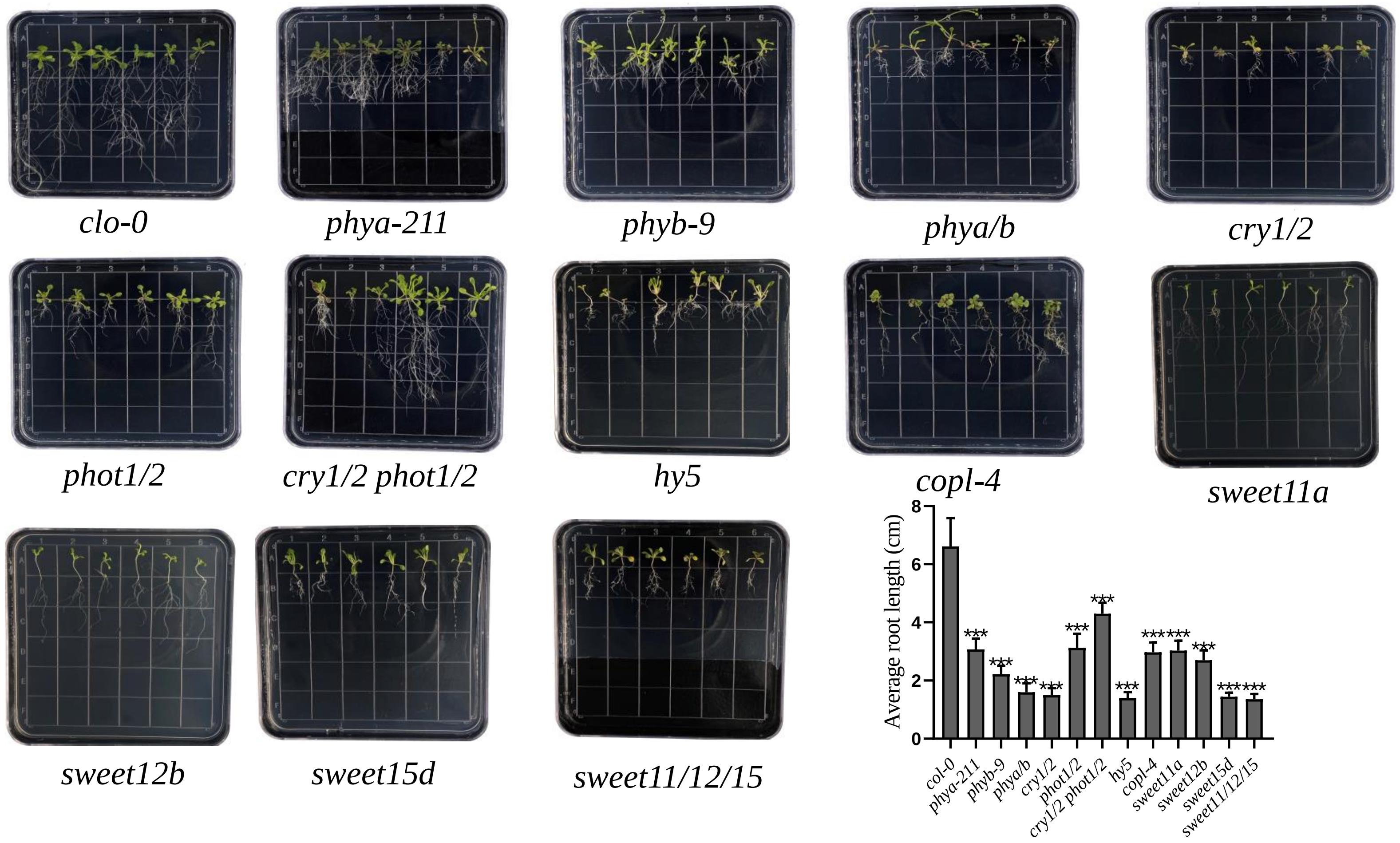

Figure S1: The photoreceptor mutants were grown on 1/2MS medium for 10 days. Root length of these mutants was compared in (b). Values are mean ± SD of more than 15 plants of each genotype. Asterisks indicate significant change of root length in various mutants as compared to that in Col-0.(*P<0.05; **P<0.01; ***P<0.001, two-tailed t-test).
