# Supplementary material for: Light signaling regulates root-knot nematode infection and development via HY5-SWEET signaling
Source: BMC Plant Biol. 2024 Jul 11;24:664. doi: 10.1186/s12870-024-05356-2 (PMC11238492; doi:10.1186/s12870-024-05356-2)
Supplement: Supplementary file 4 — Supplementary Material 4 [file 12870_2024_5356_MOESM4_ESM.pdf]

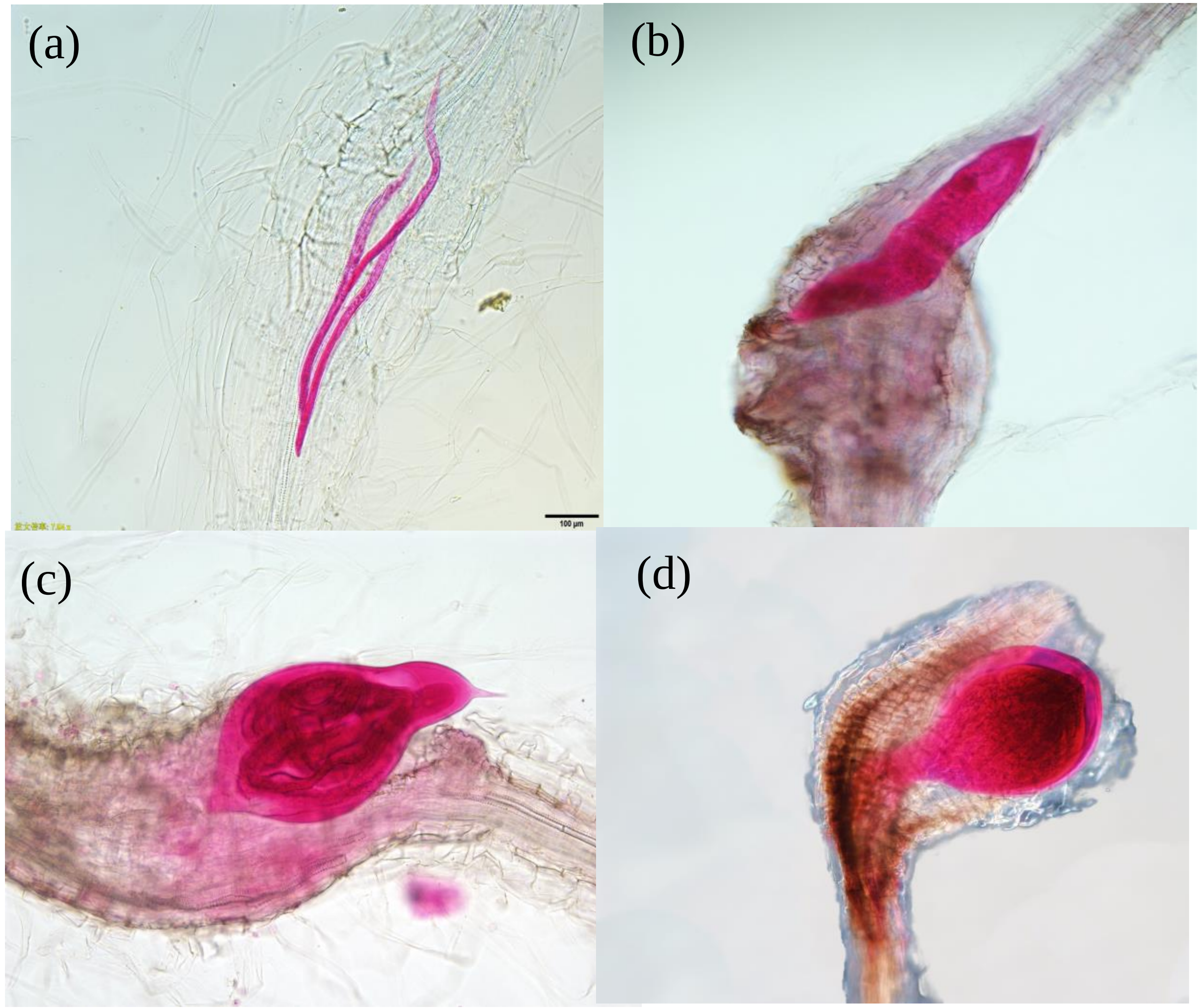

Figure S2: The Standards for counting nematodes of different developmental stages in *Arabidopsis* root tissue. (a) second-stage juveniles stage; (b) Sausage-J2 stage; (c) Globose stage; (d)adult stage. Scale bar: 100 μm.
